# Mesenchymal stromal cells donate mitochondria to articular chondrocytes exposed to mitochondrial, environmental, and mechanical stress

**DOI:** 10.1038/s41598-022-25844-5

**Published:** 2022-12-13

**Authors:** Megan Fahey, Maureen Bennett, Matthew Thomas, Kaylee Montney, Irene Vivancos-Koopman, Brenna Pugliese, Lindsay Browning, Lawrence J. Bonassar, Michelle Delco

**Affiliations:** 1 Department of Clinical Sciences, College of Veterinary Medicine, Cornell University, Ithaca, NY 14853 USA; 2 Meinig School of Biomedical Engineering, Sibley School of Mechanical and Aerospace Engineering, Cornell University, Ithaca, NY 14853 USA

**Keywords:** Cell biology, Stem cells

## Abstract

Articular cartilage has limited healing capacity and no drugs are available that can prevent or slow the development of osteoarthritis (OA) after joint injury. Mesenchymal stromal cell (MSC)-based regenerative therapies for OA are increasingly common, but questions regarding their mechanisms of action remain. Our group recently reported that although cartilage is avascular and relatively metabolically quiescent, injury induces chondrocyte mitochondrial dysfunction, driving cartilage degradation and OA. MSCs are known to rescue injured cells and improve healing by donating healthy mitochondria in highly metabolic tissues, but mitochondrial transfer has not been investigated in cartilage. Here, we demonstrate that MSCs transfer mitochondria to stressed chondrocytes in cell culture and in injured cartilage tissue. Conditions known to induce chondrocyte mitochondrial dysfunction, including stimulation with rotenone/antimycin and hyperoxia, increased transfer. MSC-chondrocyte mitochondrial transfer was blocked by non-specific and specific (connexin-43) gap-junction inhibition. When exposed to mechanically injured cartilage, MSCs localized to areas of matrix damage and extended cellular processes deep into microcracks, delivering mitochondria to chondrocytes. This work provides insights into the chemical, environmental, and mechanical conditions that can elicit MSC-chondrocyte mitochondrial transfer in vitro and in situ, and our findings suggest a new potential role for MSC-based therapeutics after cartilage injury.

## Introduction

ATP generated by mitochondrial (MT) oxidative phosphorylation is critical for cell survival and repair, and MT are the main source of reactive oxygen species in most tissues. Therefore, it is unsurprising that MT dysfunction is a fundamental pathology underlying many degenerative diseases of highly metabolic tissues, including chronic obstructive pulmonary disease^1^, degenerative retinopathy^2^, ischemic cardiomyopathy^3^, and Parkinson's^4^.

Amongst diverse tissue types, there is a spectrum of reliance on MT metabolism; cardiomyocytes heavily utilize MT oxidative phosphorylation to sustain high energy demand, while chondrocytes, the sole cell type in articular cartilage, are highly glycolytic, producing only 10–25% of their ATP by MT respiration^5,6^. Further, articular cartilage is avascular, and chondrocytes are adapted to extreme environmental conditions, including low nutrient availability and oxygen concentrations of ~ 5%^7,8^. Cartilage contains at least an order of magnitude fewer cells per tissue volume and only ~ 5% of the MT volume per cell compared to the liver^6^. Taken together, this evidence would seem to minimize the importance of MT in cartilage health and disease and may explain why the role of MT dysfunction in the initiation and pathogenesis of osteoarthritis (OA) has not been well studied. Recent work by our group and others revealed that MT dysfunction is one of the earliest responses of cartilage to injury, resulting in cell death, degeneration of the extracellular matrix, and ultimately post-traumatic OA^9,10^. We also found that early pharmacological intervention using the MT protective peptide SS-31 after cartilage injury reduced chondrocyte death and prevented cartilage degeneration^11–14^. While these studies provide rationale for targeting MT function to prevent OA and similar degenerative diseases of avascular tissues, strategies for restoring MT function to improve tissue healing have not been developed.

Mesenchymal stromal cells (MSCs) are being widely investigated as regenerative therapies, and increasing evidence suggests MSCs can relieve symptoms of OA including pain and joint dysfunction, as well as preserve cartilage^15,16^. While the mechanisms governing the beneficial effects of implanted cells remain unclear, contemporary evidence indicates that MSCs act mainly by modulating the joint environment via their secreted products, which inhibit catabolic signaling pathways and promote anabolic responses^17^.

Intriguingly, recent evidence suggests an alternate paradigm; MSCs can rescue injured cells undergoing MT dysfunction by donating functional MT. This intercellular MT transfer restores bioenergetics and preserves viability of recipient cells under metabolic stress^18^. In vitro, MT transfer was evident within 12 h of co-culture between MSCs and vascular endothelial cells subjected to ischemia–reperfusion injury^19^. In an in vivo model of acute lung injury from endotoxin installation into the airways of mice, MT transfer from MSCs to alveolar epithelial cells increased ATP concentration^20^. These and other studies of MT transfer have involved oxidative cell types, such as cardiomyocytes^3^ and neurons^21^. While chondrocytes are not considered oxidative, the role MT dysfunction in OA supports investigating MT transfer in cartilage. Wang et al. (2021) reported evidence of MT transfer to chondrocytes in cell culture using fluorescent imaging and that MT transfer is associated with improvement of MT function^22^; however, MT transfer was not confirmed or quantified, and the mechanisms of transfer have not been investigated. Although the specific mechanisms of intercellular MT transfer are still largely unknown, several processes have been implicated in MT transfer between MSCs and dissimilar cell types, including tunneling nanotubule formation, gap junction signaling, extracellular vesicle communication, and cell–cell fusion events^23–25^. Importantly, several models have shown transfer of MT in a connexin 43 (Cx43)-dependent manner^20,26^. Cx43 is the protein monomer of gap junctions that connect adjacent cells and is known to be expressed by chondrocytes^27^. Notably, chondrocyte Cx43 expression is increased in OA^28^ and when stressed with IL-1ß^29^.

Current literature suggests that implanted MSCs can be recruited to damaged tissues by cells undergoing metabolic stress and establish gap junctions to donate MT^30,31^. However, this has not been investigated in cartilage. Therefore, our goal was to develop model systems to study MT transfer from MSCs to chondrocytes, study factors that increase MT transfer, and begin to identify the mechanisms involved. We hypothesized that MT transfer from MSCs to chondrocytes would occur in vitro and in situ, factors that inhibit chondrocyte MT function would increase MT transfer from MSCs, and that inhibiting gap junctions, specifically Cx43, would decrease transfer. Identifying mechanisms that mediate MT transfer in cartilage is the first step toward developing approaches to exploit this biological phenomenon to improve healing in avascular orthopedic tissues.

## Results

### MSCs transfer MT to chondrocytes in vitro

**Figure 1:**
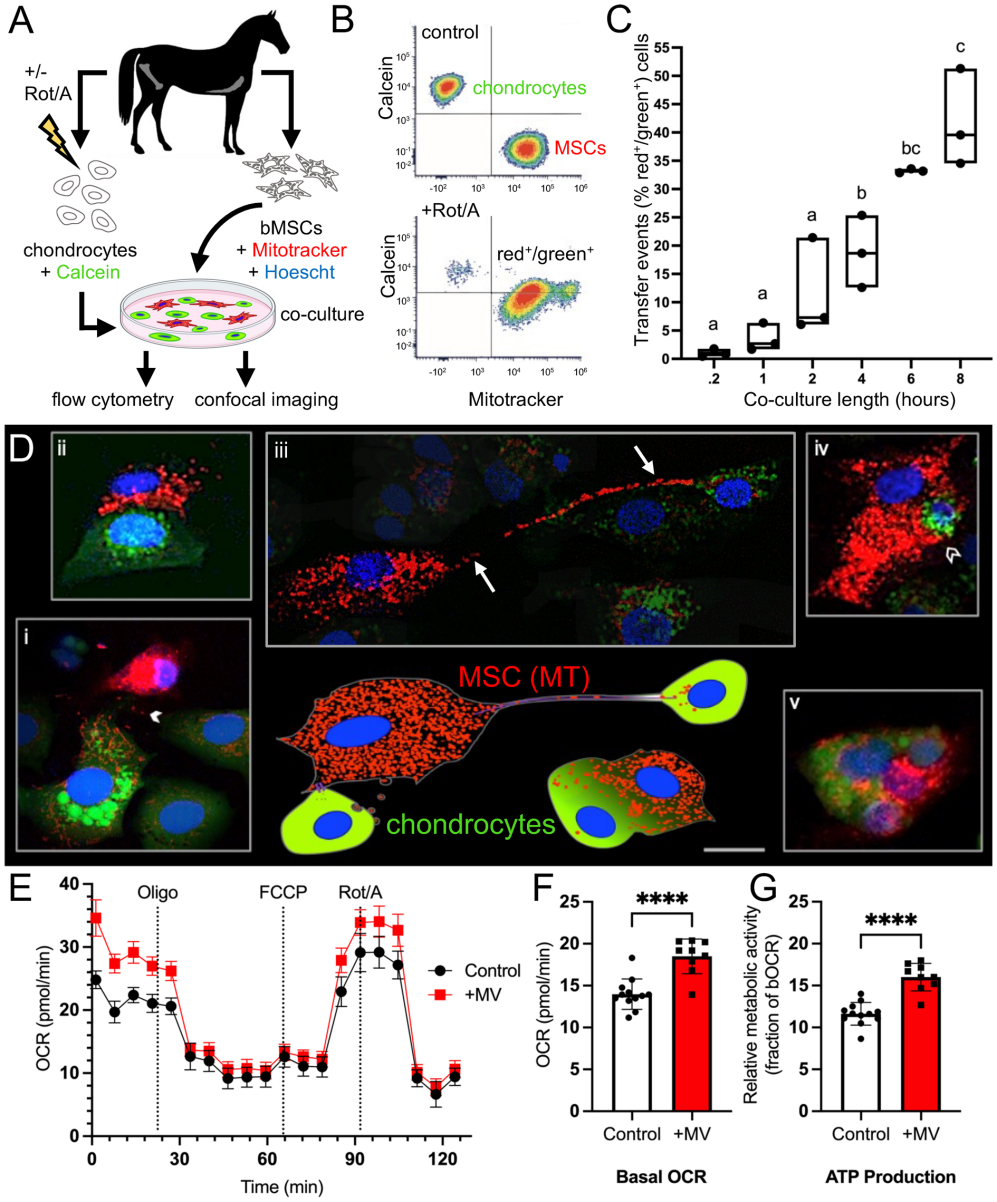
Equine mesenchymal stromal cells (MSCs) transfer mitochondria (MT) to chondrocytes over time and via several modes and increase MT function. (**A**) Experimental design (**B**) Representative flow cytometry plots. Top plot shows results of separately cultured equine chondrocytes stained with Calcein AM (4 mM) and MSCs stained with MitoTracker Deep Red (200 nM) used to set quadrant gates; bottom plot shows red^+^/green^+^ chondrocytes (upper right-hand quadrant) after rotenone and antimycin (Rot/A; 0.5 μM/0.5 μM) stimulation and co-culture with MSCs. (**C**) MT transfer (% red^+^/green^+^ cells) increases with length of co-culture, n = 3. (**D**) Schematic (bottom center) depicting observed modes of MT transfer from MSCs (red MT, blue nuclei) to chondrocytes (green cytoplasm, blue nuclei) alongside corresponding confocal microscopy images (i-v), taken up to 8 h after initiation of co-culture, stained as above with the addition of Hoechst (5 mg/L, blue) to MSCs before co-culture; representative images of (i) likely gap junction-mediated microvesicle (MV, arrowhead) transfer, (ii) close cellular contact without apparent transfer demonstrating the broad range of observed cell–cell interactions, (iii) possible tunneling nanotubule-mediated filopodial (arrows) transfer, (iv) apparent phagocytosis-like event (open arrowhead), (v) cell fusion event. Note that in (i) and (iii), contrast in surrounding cells was decreased 10–20% to highlight the mentioned interaction; Bar ≅ 10 µm. (**E**) Equine chondrocyte oxygen consumption rate (OCR) during real-time microscale respirometry (Seahorse MT stress test) with and without MV treatment. (**F**) Respirometry data shows MV treatment increases equine chondrocyte basal OCR. (**G**) ATP production in equine chondrocytes increases with MV treatment.

To determine if MT transfer occurs between MSCs and chondrocytes, a primary cell co-culture model was used; equine articular chondrocytes and MSCs were harvested, cultured, and stained with Calcein AM or MitoTracker Deep Red and Hoescht, respectively. Chondrocytes were stressed with MT-specific inhibitors rotenone and antimycin (Rot/A), then co-cultured with MSCs for up to 8 h at physiologic oxygen concentration for chondrocytes (5% O_2_; Fig. 1A). Flow cytometry was used to quantify MT transfer events; separately cultured chondrocytes and MSCs were used to set up quadrant gates that identified chondrocytes by green fluorescence and MSCs by red fluorescence (Fig. 1B; top plot). This gating strategy was used on co-cultured cells, which allowed us to quantify the upper right-hand quadrant of cells positive for both stains (red^+^/green^+^ cells), identifying what we considered transfer events (Fig. 1B; bottom plot). The percentage of red^+^/green^+^ cells increased with duration of co-culture up to 8 h (Fig. 1C). Confocal microscopy confirmed that stimulated chondrocytes gained red MT fluorescence, and MSCs gained green fluorescence, indicative of intercellular MT transfer. Several modes of transfer were visualized including apparent extracellular vesicle-mediated transfer (Fig. 1Di), with and without evidence of cell–cell contact (Fig. 1Dii), tunneling nanotubule-mediated filopodial transfer (Fig. 1Diii), and cell fusion events (Fig. 1Div,v). We investigated the effects of transfer on chondrocyte function using non-contact microvesicle (MV)-mediated transfer as recently reported^32^. Primary equine chondrocytes cultured under hyperoxic conditions (21% O_2_) were treated with MSC-derived MVs for 12 h. Treated chondrocytes had increased basal oxygen consumption rate (bOCR) and ATP production compared to untreated controls, indicating improved MT respiratory function (Fig. 1E–G, see Supplemental Fig. S2 for additional outcome measures). There are limitations associated with live cell staining techniques including MT toxicity from stains, short-term fluorescence, and rapid photobleaching. We also observed the indirect staining of equine chondrocytes with Hoescht from co-culture with the Hoescht-stained MSCs (Fig. 1D). Given these limitations, we further investigated intercellular MT transfer using transgenic mouse strains expressing endogenous fluorescent proteins.

**Figure 2:**
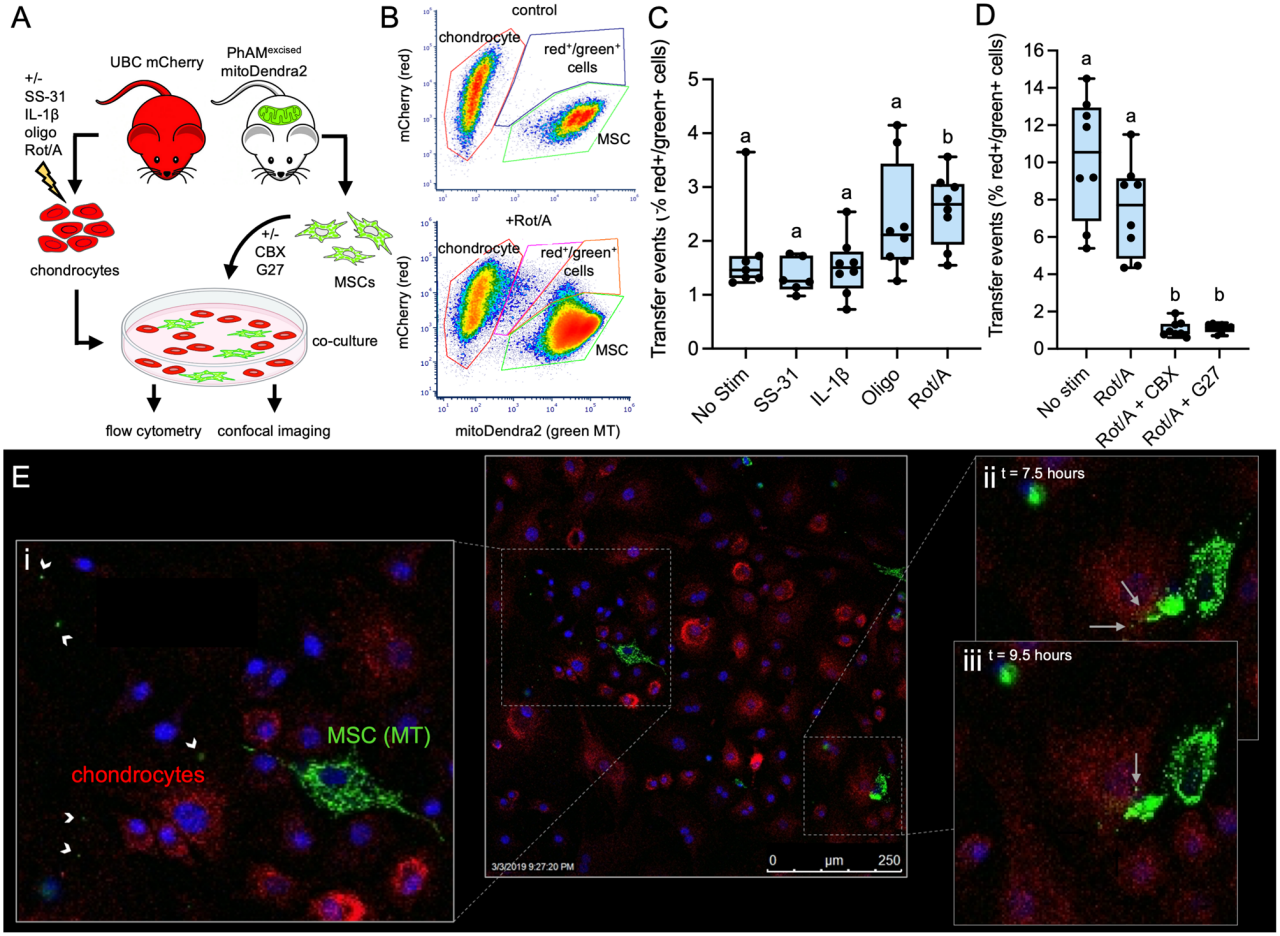
Murine mesenchymal stem cells (MSCs) donate mitochondria (MT) to chondrocytes and transfer is increased by MT-specific stimulants and decreased with gap-junction inhibitors. (**A**) Experimental design (**B**) Representative flow cytometry plots. Top plot shows results of separately cultured mitoDendra2 MSCs and mCherry chondrocytes used to set gates; bottom plot shows chondrocytes co-cultured with MSCs after stimulation with Rotenone and Antimycin (Rot/A; 0.5 μM/0.5 μM). (**C**) MT transfer events (% red^+^/green^+^ cells) from MSCs to chondrocytes after 12 h co-culture is significantly higher when chondrocytes are stimulated with the MT-specific stressor Rot/A under physiologic culture conditions (5% O_2_, 0.45 g/L glucose), n = 4. (**D**) Both carboxonolone disodium (CBX; 100 μM) and gap 27 (G27; 100 μM) significantly inhibited MT transfer events. MSCs were treated with these gap junction inhibitors during 24 h co-culture, n = 4 (**E**) Live confocal microscopy of MT transfer. MSCs were stained with Hoechst (5 mg/L) for visualization of nuclei. (i) Apparent MSC microvesicles containing MT (white arrowheads); image acquired 4.5 h after initiation of co-culture, (ii) same position 3 h later, MSC MT transferred to chondrocyte (white arrows) (iii) same position 5 h later. Co-culture was conducted in growth medium on Leica SP5 inverted confocal microscope outfitted with temperature and humidity-controlled chamber, using a 40 × objective lens. Scale bar = 250 μm.

Articular chondrocytes were harvested and cultured from UBC mCherry mice, with ubiquitous red fluorescent cytoplasmic protein expression, and MSCs were isolated and expanded from PhAM mitoDendra2 mice, which express a MT-targeted green fluorescent protein. Co-culture of these cell types allowed flow cytometry and confocal imaging to confirm intercellular MT transfer (Fig. 2A). Longitudinal visualization of cellular interactions over 9.5 h of co-culture revealed MSCs shedding green MT (Fig. 2Ei; white arrowheads) and localization of green MT in red chondrocytes at 7.5 (Fig. 2Eii) and 9.5 h (Fig. 2Eiii) of co-culture. The full time-lapse can be viewed in the supplement (Movie S1).

### Inhibition of MT respiration affects MT transfer events

We investigated if MT dysfunction in chondrocytes is associated with increased intercellular MT transfer in our transgenic murine model. Chondrocytes were cultured under physiologic oxygen and glucose concentrations (5% O_2_ and 0.45 g/L glucose), stimulated with either a general inflammatory stimulus (IL-1ß) or MT-specific inhibitors (Rot/A or oligomycin (Oligo)) prior to co-culture with MSCs, and evaluated using flow cytometry. Because 2-dimensional culture alone is a known stressor in chondrocytes, as an additional negative control chondrocytes were treated with SS-31, a MT-protective peptide that improves ATP turnover and prevents proton leak in chondrocytes^11^. Gates for red^+^ and green^+^ cells were created based on non-co-cultured controls (Fig. 2B; top plot). These gates were used to quantify red^+^/green^+^ cells after treatments and co-culture with MSCs (Fig. 2B; bottom plot). We found that inducing MT dysfunction by inhibiting the electron transport chain with Rot/A increased the percentage of red^+^/green^+^ cells compared to the other stimulants and non-stimulated control (Fig. 2C). The ratio of chondrocytes to MSC in this experiment was 10:1. When the ratio was altered to 2:1, Rot/A did not affect transfer events but the percentage of red^+^/green^+^ cells was higher in both non-stimulated and Rot/A stimulated chondrocytes (Fig. 2D). Pre-treatment with SS-31 limited the rate of MT transfer to < 2% but this was not significantly different from the non-stimulated control group (Fig. 2C).

### Gap junction inhibition prevents MSC-chondrocyte MT transfer

Gap junctions allow physical interaction and direct signaling between dissimilar cell types, and have been implicated in MSC MT donation^31^. We investigated this mechanism in our co-culture system by treating murine chondrocytes and MSCs with gap junction inhibitors and assessing their effect on the percentage of red^+^/green^+^ cells. Carbenoxolone disodium (CBX) is a derivative of 18-alpha-glycyrrhetinic acid and a non-specific inhibitor of gap junction electrical coupling^33^. Treatment with CBX reduced the percentage of red^+^/green^+^ cells by sevenfold (Fig. 2D). A more specific inhibitor, Gap 27 (G27), was used to reversibly inhibit Cx43-mediated cell–cell communication^34^, which prevented MT transfer to a similar extent as CBX (Fig. 2D).

### Culture conditions affect MSC-chondrocyte MT transfer

**Figure 3:**
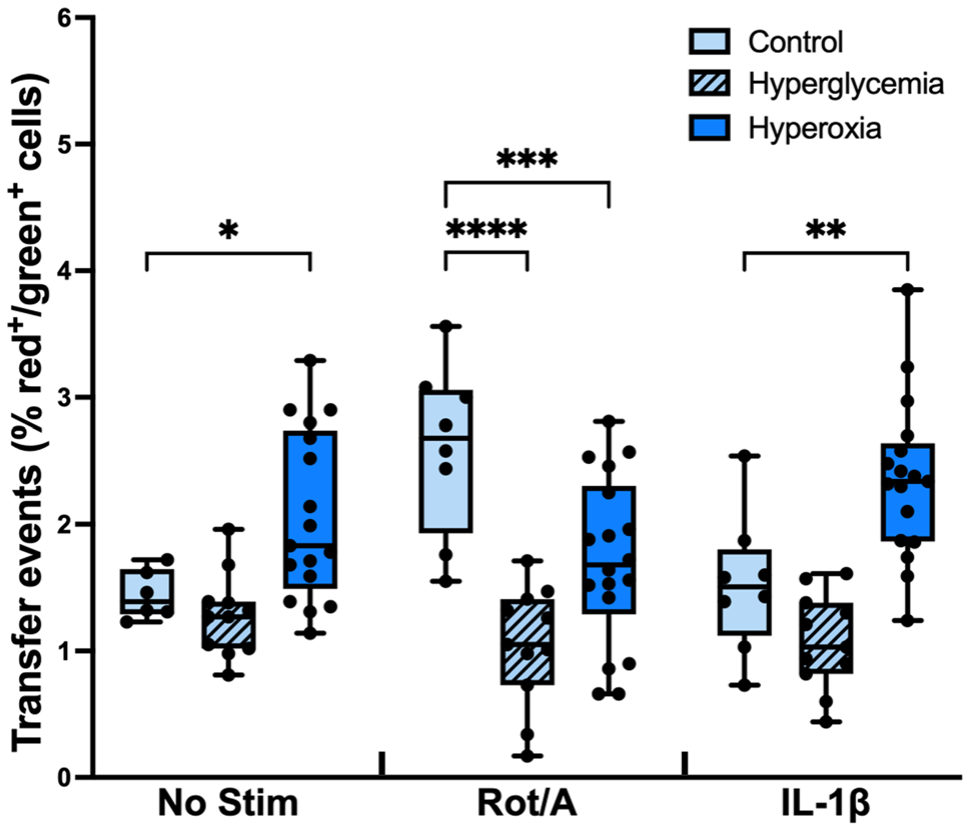
MSC-chondrocyte MT transfer is affected by non-physiologic culture conditions. Murine mCherry chondrocytes (red) and PhAM mitoDendra2 MSCs (green MT) were co-cultured in hyperoxia or hyperglycemia for 12 h after stimulation with or without Rot/A or IL-1β. MT transfer events (% red^+^/green^+^ cells) were quantified by flow cytometry. Hyperoxia increases transfer events in non-stimulated and IL-1β-stimulated chondrocytes (*P* < 0.05). Hyperoxia and hyperglycemia decreased transfer events when chondrocytes were stimulated with Rot/A (*P* < 0.0007). n = 4.

Because homeostatic chondrocytes are adapted to low oxygen and nutrients conditions in vivo, we evaluated the effects of environmental culture conditions on MT transfer. Murine chondrocytes were isolated and cultured at physioxia (5% O_2_) or relative hyperoxia (21% O_2_), and euglycemia (0.45 g/L) or hyperglycemia (1 g/L), then stressed with Rot/A or IL-1ß. The percentage of red^+^/green^+^ cells was determined by flow cytometry as previously described. Non-physiologic culture conditions alone, and their interaction with stimulation, affected MT transfer events (Fig. 3; *p* < 0.0001).

Hyperoxia increased MT transfer in both non-stimulated and IL-1ß-stimulated chondrocytes (*p* < 0.0017). Hyperglycemia caused MT transfer to decrease in Rot/A chondrocytes relative to Rot/A chondrocytes in physiologic conditions. When chondrocytes were non-stimulated or stimulated with IL-1ß, hyperglycemia did not affect transfer.

### Hyperoxia increases chondrocyte expression of gap junction-, inflammation-, and senescence-associated genes

**Figure 4:**
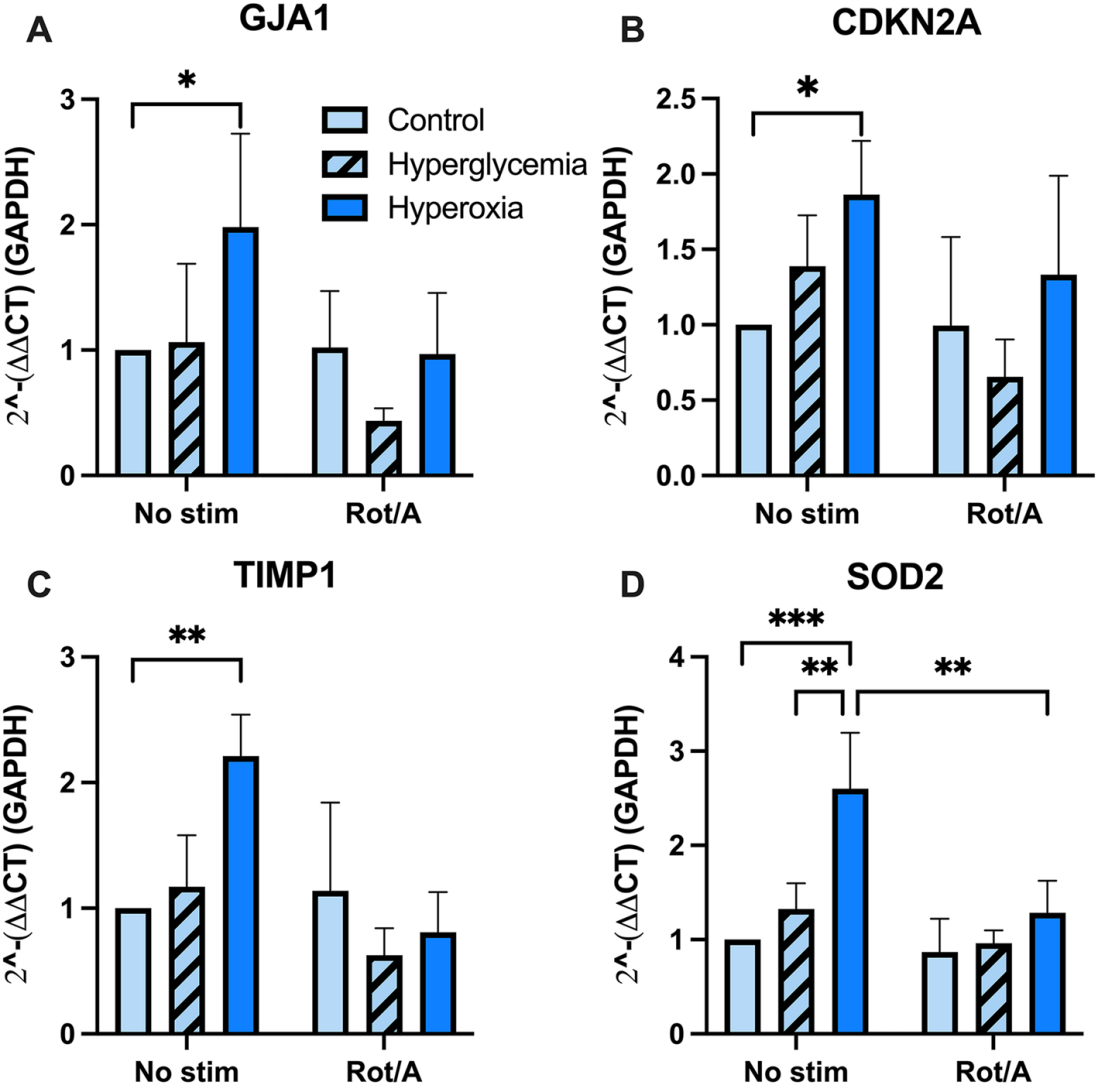
Chondrocyte gene expression changes in hyperoxia and with rotenone/antimycin inhibition. (**A**) Gap junction protein alpha 1 (GJA1) expression increased in non-stimulated chondrocytes cultured in hyperoxia, n = 4. (**B**) Cycling dependent kinase inhibitor 2A (CDKN2A) expression increased in hyperoxia, n = 4. (**C**) Tissue metallopeptidase inhibitor 1 (TIMP1) expression increased in non-stimulated chondrocytes cultured in hyperoxia, n = 4. (**D**) Superoxide dismutase 2 (SOD2) expression increased in chondrocytes cultured in hyperoxia, n = 4.

To begin to understand the link between increased MT transfer and MT dysfunction in chondrocytes, we developed a custom quantitative PCR panel of relevant genes involved in chondrocyte metabolism and OA. We found that expression of gap junction alpha 1 (GJA1), the gene that encodes the Cx43 protein, was increased in hyperoxia (Fig. 4A). The senescence associated markers, cyclin-dependent kinase inhibitor 2A (CDKN2A) and tissue inhibitor of metalloproteinases 1 (TIMP1), were also increased in hyperoxia (Fig. 4B, C). In addition, hyperoxia caused chondrocytes to increase the expression of the antioxidant enzyme, superoxide dismutase (SOD2; Fig. 4D).

### MSCs transfer MT to chondrocytes in injured cartilage explants

**Figure 5:**
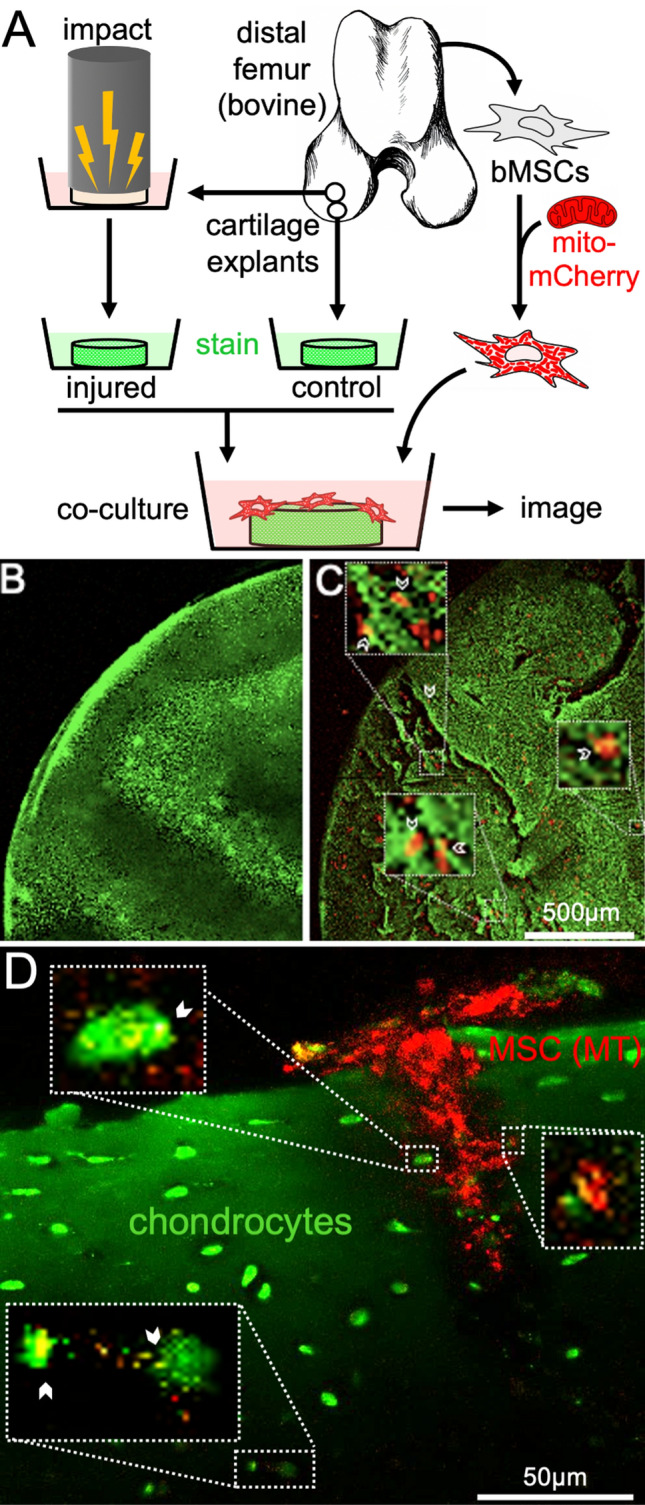
Bovine mesenchymal stromal cells (MSCs) home to damaged cartilage and transfer mitochondria (MT) to chondrocytes in situ. (**A**) Schematic depicting experimental design for cartilage explant injury. (**B**) Representative image of unimpacted cartilage explant stained with carboxyfluorescein diacetate (green) and seeded with lentiviral transduced mCherry-mito MSCs (red MT) compared to (**C**) injured cartilage seeded with MSCs and cultured for 4 days. MSCs localized to damaged areas on injured cartilage, while MSCs were not present on the surface of uninjured cartilage. (**D**) High magnification images of MSC (red MT) localized deep within a crack in injured cartilage (articular surface is toward the top). MSC interactions with chondrocytes (green) are evident in several locations (white arrowheads) and MT transfer is evident in areas of red^+^/green^+^ co-localization (yellow) close to the crack (top insets) as well as remote and deep to the crack (bottom inset).

To assess whether MSCs can deliver MT to chondrocytes embedded in cartilage tissue in situ, we utilized an established model of cartilage injury in which cartilage explants are impacted at loading rates capable of inducing extracellular matrix damage and MT dysfunction^5,35,36^. Bovine cartilage explants were injured and stained with carboxyfluorescein diacetate, a green cellular dye, then incubated with bovine MSCs transduced with mCherry-mito, a MT-targeted red fluorescent protein, for 4 days (Fig. 5A). Confocal imaging revealed that MSCs adhered to the articular surface and localized to areas of matrix damage in injured explants (Fig. 5C). In contrast, MSCs were rarely identified on the surface of uninjured cartilage (Fig. 5B). Cross-sectional confocal imaging of injured explants (Fig. 5D) revealed that MSCs extended into cracks in the cartilage matrix to depths of more than 100 µm from the articular surface. MSC MT co-localized with chondrocytes near microcracks and in direct contact with MSCs. Deeper in the tissue, MSC MT were identified within chondrocytes at > 50 µm from cracks, with no evidence of physical interaction between chondrocytes and MSCs. In addition to MSC-chondrocyte transfer, murine MSCs injected into the femorotibial (knee) joint of mCherry mice also localized to the synovial membrane and appeared to donate MT to synoviocytes (Supplemental Fig. S1).

## Discussion

This study provides evidence of intercellular MT transfer from bone marrow derived MSCs to articular chondrocytes in cell culture and in injured cartilage tissue. The phenomenon of MT donation by MSCs has primarily been described in energy-expensive tissues, such as heart and brain, which heavily utilize MT oxidative phosphorylation for ATP production^3,21^. Although healthy cartilage derives the majority of its energy from glycolysis, MT function is critical to chondrocyte homeostasis and extracellular matrix (ECM) synthesis^5^. The highly ordered structure of the ECM endows articular cartilage unique functional properties of cushioning and lubricating joints. Notably, mice with impaired MT electron transport chain proteins had altered ECM composition, resulting in increased cartilage stiffness^37^. MT are highly plastic organelles that undergo morphological and functional changes in response to synthetic and bioenergetic demands^38^. However, recent work revealed that in chondrocytes, MT polarity and oxygen consumption rate per viable cell decreases in the hours following mechanical injury^9,14^. This suggests that with few MT, the ability of chondrocytes to upregulate ATP production and biosynthetic capacity in the critical period following injury is limited. While chondrocytes can increase MT content via biogenesis, this requires time (hours-days) and an initial energy investment. Taken together, this evidence suggests that while MT diseases and MT-targeted therapies are generally associated with metabolically active, highly oxidative tissues, cells with minimal spare respiratory capacity such as chondrocytes may recruit MT donation as a rescue mechanism during times acute of stress. Further studies are necessary to investigate the functional impact of MT transfer on cartilage.

MT dysfunction has recently been identified as a key subcellular event in the initiation and early pathogenesis of OA^39,40^. Although the mechanisms are unclear, MT dysfunction in recipient cells appears to be a prerequisite for the initiation of MT donation by MSCs in other cell types^18^, and our findings support this in chondrocytes. We investigated various stimuli known to induce MT dysfunction. IL-1ß is a well-established in vitro model of OA, and this ‘master’ pro-inflammatory cytokine has been found to play a central role in disease pathogenesis^41^. Further, inflammatory cytokines including IL-1ß and TNF-alpha induce chondrocyte MT dysfunction by indirectly inhibiting the activity of MT Complex I^42^. While IL-1ß did not affect MT transfer in our murine co-culture system, a more specific MT inhibitor, Rot/A increased transfer events. Rot/A inhibits MT complexes I and III, respectively. By inhibiting the flow of electrons through the electron transport chain, Rot/A prevents ATP production while also increasing reactive oxygen species due to incomplete transfer of electrons to oxygen in the final step of oxidative phosphorylation^43^. Our findings suggest that bioenergetic and/or oxidative stress in chondrocytes may induce MT transfer. Conversely, we found that Oligo, which blocks ATP synthase, did not increase transfer events. Since complexes I and III are the major sites of MT reactive oxygen species production^5^, these findings suggest oxidative stress may be more important than bioenergetic stress in eliciting intracellular transfer in this model. Our work quantified transfer events up to 24 of co-culture. Future experiments will aim to elucidate the time-dependency of MT transfer from MSCs to chondrocytes with and without stimulation.

Non-physiologic oxygen and nutrient conditions are known to affect chondrocyte metabolism. Of note, relative hyperoxia (21% O_2_) and hyperglycemia (1 g/L glucose) are considered standard conditions for culturing articular chondrocytes, but are not representative of the low nutrient availability and avascular nature of cartilage^5^. Therefore, we compared transfer events to chondrocytes at physioxia (5% O_2_) versus relative hyperoxia (21% O_2_) and euglycemia (0.45 g/L glucose) versus relative hyperglycemia (1.0 g/L glucose). As expected, we found that hyperoxia caused an increase in transfer events in both non-stimulated and IL-1ß-stimulated chondrocytes, supporting our hypothesis that hyperoxia-induced MT dysfunction triggers MT transfer. Under these same conditions, the expression of the MT-specific antioxidant SOD2, was increased. This is consistent with literature demonstrating that chondrocytes cultured in hyperoxia experience oxidative stress due to increased superoxide production^5^. Taken together, these data suggest oxidative stress caused by hyperoxia can induce MT transfer. Interestingly, chronic MT dysfunction in end-stage OA has been associated with decreased SOD2; OA chondrocytes from patients undergoing hip replacement surgery demonstrate SOD2 deficiency, which has been causally linked to oxidative damage mediated by MT-derived reactive oxygen species^39,40^.

In further support of the hypothesis that MT-derived reactive oxygen species triggers MT transfer from MSCs, chondrocytes increased expression of inflammation- and senescence-associated genes in hyperoxic culture; Increased TIMP1 expression, a regulator of metalloproteinases and ECM turnover, is indicative of an inflammatory phenotype in chondrocytes^44^. We also found that CDKN2A, a marker of chondrocyte senescence^45^, was increased in hyperoxia. These findings are in agreement with studies that reveal excess MT-derived reactive oxygen species upregulate release of inflammatory cytokines including IL-1ß, cartilage ECM-degrading enzymes matrix metalloproteinase 1 and 3, and senescence-associated β-galactosidase in part through activation of the NF-kB signaling pathway in chondrocytes^45–48^.

In other tissue types, Cx43 and other gap junction proteins have been found to play a critical role in mediating intercellular MT transfer^20,26^. In an endotoxin model of acute lung injury, Cx43 was required for MSCs to attach to alveoli and generate Cx43-positive nanotubules and extracellular vesicles, which resulted in MT transfer and increased intracellular ATP in recipient cells^20^. We found that co-cultures treated with the Cx43-specifc inhibitor Gap27 had a sevenfold decrease in transfer events. Gap27 is a small, synthetic mimetic peptide that reversibly inhibits the channel function of Cx43^34^. This suggests that Cx43-based MSC-chondrocyte signaling is required for MT transfer in our model. Interestingly, Mayan et al.^28^ found that chondrocyte GJA1 (the gene encoding Cx43) expression and protein content was increased in OA cartilage. Here, we report that hyperoxic stress increases both MT transfer and GJA1 expression in chondrocytes. On confocal imaging of co-cultures, we observed several modes of transfer associated with connexin signaling in other tissues, including apparent tunneling nanotubule mediated transfer. Induced pluripotent stem cell (iPSC)-derived MSCs donate MT via tunneling nanotubules in a Cx43-dependent manner^26^. In the current study, we investigated bone marrow derived MSCs as a vehicle for MT transfer because this cell source is commonly used in clinical treatment of OA^16^. Interestingly, several studies have shown that iPSC-MSCs exhibits a higher frequency of MT donation than bone marrow derived MSCs under specific conditions^49–51^, suggesting iPSC-MSCs may be an alternative source for MT transfer in OA treatment. Future studies would be needed to compare the efficacy and clinical feasibility of iPSCs versus bone marrow derived MSCs for MT transfer in cartilage. In addition, further studies are necessary to elucidate specific mechanisms of MSC-chondrocyte MT transfer, and determine which processes predominate under specific conditions, including naturally occurring OA.

While MT transfer has been documented in several highly cellular tissues, a major potential barrier to MT transfer in articular cartilage is the low cellularity and high density of the ECM; The small pore size and highly charged nature of the cartilage ECM has been shown to inhibit transport of large particles such as antibodies and other investigational biologic therapies^52,53^. Therefore, in order to investigate the relevance of MSC-chondrocyte MT transfer in situ, we utilized a well-validated model of cartilage impact injury^9^. Following injury, MSCs localized to areas of cartilage matrix damage, and donated MT to adjacent chondrocytes, confirmed by 3-dimensional co-localization of MSC MT fluorescent signal within chondrocytes. The striking frequency of direct MSC-chondrocyte interactions at sites of matrix damage suggests a signaling pathway, whereby MSCs home to and/or chondrocytes recruit MSCs to areas of tissue damage to initiate transfer. This is supported by literature that implicates reactive oxygen species, TNFα, and NFκB signaling in triggering MT transfer from MSCs to other cell types^18^. Further studies are necessary to investigate such mechanisms in cartilage.

Cross-sectional imaging of injured explants revealed evidence of close cellular interactions and MT transfer from MSCs to chondrocytes located immediately adjacent to microcracks in the cartilage ECM. We also identified recipient chondrocytes greater than 50 µm from crack interfaces. Previous studies have documented MT transfer via extracellular vesicles^18,32^. As such, the finding of apparent non-contact MT transfer in cartilage may be a result of MSCs exporting MT in extracellular vesicles which are taken up by chondrocytes embedded within distant lacunae. This is supported by our in vitro longitudinal imaging studies, where MSCs appeared to shed MT into the extracellular environment. However, the highly charged ECM of cartilage may present a barrier to passive diffusion of these large, membrane-bound particles. Alternatively, it is possible that our in-situ imaging studies failed to detect limited sites of MSC-chondrocyte contact. Recent evidence suggests that, contrary to conventional wisdom, chondrocytes are not isolated within lacunae but rather connected to one another by long, thin filapodial arms that extend through the ECM^27^. In that study, Cx43-based gap junctions were found to be enriched at sites of filopodial contact. Given our findings implicating Cx43 in MSC-chondrocyte MT transfer, further investigation into gap junction signaling between MSCs and in situ chondrocytes is warranted.

In summary, this work provides quantitative and qualitative evidence of intracellular MT transfer from MSCs to articular chondrocytes. We demonstrated that inhibition of Cx43 based gap junction signaling prevents MSC-chondrocyte MT transfer. Importantly, we also documented MT transfer to mechanically injured chondrocytes embedded within the native cartilage matrix. Although MT transfer has been documented in vivo in other tissues^20^, further studies are warranted in live animal models to address MT transfer in cartilage. However, this study presents multiple in vitro and ex vivo models that may be used to investigate MT transfer as a novel biologic approach to augment MT capacity in chondrocytes and other poorly healing tissues of the skeletal system.

## Materials and methods

### Equine cell co-culture model

Primary chondrocytes were previously harvested from normal femoropatellar joints of young-adult horses (2–5 years, n = 3) that were euthanized for reasons unrelated to this study. Chondrocytes were cultured on T175 plates with Ham’s F12 media (Corning, 1X), with 10% fetal bovine serum (R&D Systems), 1% MEM Amino Acids (gibco, 50X), 2.5% HEPES (Corning, 1M), 1% Penicillin–Streptomycin (Corning, 100X) in 5% CO_2_, 21% O_2_ at 37 °C. Bone marrow-derived MSCs were also isolated and cultured on T175 plates with Dulbecco’s Modified Eagle Medium (DMEM) with 1 g/L glucose (Corning, 1X) media with 10% fetal bovine serum (R&D systems), 1% Penicillin–Streptomycin (Corning, 100X), and 0.5% basic fibroblast growth factor (Corning, 1 µg/mL) in 5% CO_2_, 21% O_2_. Passage 2 chondrocytes were plated on 12-well cell culture plates (250,000 cells per well) for flow cytometry and a 6-well chambered cover glass slide for confocal imaging (60,000 cells per well). At 80% confluence, chondrocytes were stressed by the addition of Rot/A (Sigma Aldrich, 0.5 μM, VWR International, 0.5 μM). After 12 h of stimulation, chondrocytes were rinsed thrice with 1% PBS, fresh chondrocyte media was replaced, and chondrocytes were stained with 1 μL of a cytoplasmic green dye, Calcein AM (Thermofisher, 4 mM). Passage 2 MSCs were stained with MitoTracker Deep Red (Thermofisher, 200 nM), a red dye targeted to intact MT, in a 50 mL conical tube with PBS. The MSCs used in the confocal imaging study were also stained with Hoechst 33342 (Thermofisher, 5 mg/L) in the same 50 mL conical tube. The MSCs were incubated in the stain(s) for 30 min and rinsed thrice with PBS to prevent residual dyes in the supernatant. The MSCs were then added to wells in a 5:1 chondrocyte:MSC ratio with equal parts chondrocyte and MSC media. The 12-well plates (n = 3) and chambered cover glass slide (n = 1) were incubated for 8 h. At pre-determined time points (10 min, 1, 2, 4, 6, 8 h), one co-culture well from each of the 12-well plates was lifted with 0.25% Trypsin–EDTA (Corning), fixed with 1% formalin, and stored in PBS at 4 °C. At the same time points, 1% formalin was directly added to one well of the 6-well chambered cover glass slide. For Seahorse experiments, chondrocytes were thawed and plated onto a 96 well microplate (Seahorse Biosciences) at a density of 35,000 cells per well and cultured as above. Equine MSCs we plated on 2 T175 flasks at a density of 1.5 × 10^6^ cells and cultured as above. At confluency, MSCs were rinsed with PBS and cultured in equine MSC media without serum for 12 h to collect conditioned media. MVs were harvested from conditioned media as previously described^32,54^. Briefly, MSC conditioned media was filtered through 0.22 µm Steriflip filter (Millipore) at a drip rate of 1 per/second. MVs captured by the filter were resuspended in PBS (~ 200 µL) and stored on ice until MV treatment. Chondrocytes were washed with PBS and switched to equine chondrocyte media without serum for 12 h to equilibrate to serum free conditions. After 12 h, media was replaced with fresh serum free chondrocyte media. Serum free chondrocyte media containing 16 µL/well MV solution was added to each treatment well. Following a 12 h incubation, media in all wells was changed to microscale respirometry assay media (DMEM containing glucose 10 mM, pyruvate 1 mM, L-glutamine 2 mM; Seahorse Biosciences) and the MT stress test was performed. Oxygen consumption rate (OCR) and extracellular acidification rate (ECAR) were measured for each well approximately every 7 min for ~ 130 min total. Following baseline respiration measurements, a MT stress test was performed using the XF Cell Mito Stress Test Kit (Seahorse Biosciences) according to previously optimized protocols; OCR and ECAR were measured in response to the addition of the following (i) Oligo (1.5 µM; 8 measurements) (ii) carbonyl cyanide-4-(trifluromethoxy) phenylhydrazone (FCCP; 2.0 µM; 4 measurements), a proton circuit uncoupler, and iii) Rot/A (0.5 µM/0.5 µM; 3 measurements). MT respiration functional indices were calculated as previously described^55^: non-MT respiration (NMR) = Rot/A-stimulated OCR; basal OCR (bOCR) = initial OCR–NMR; maximal OCR (mOCR) = FCCP-stimulated OCR–NMR; spare respiratory capacity (SRC) = mOCR–bOCR; proton leak (Oligo-stimulated OCR–NMR)/bOCR; ATP Production = (initial OCR–Oligo-stimulated OCR)/bOCR.

### Murine cell co-culture model

UBC mCherry (Jax stock 017614) and PHaM mitoDendra2 (Jax stock 018385) mice were obtained from the Michael Kotlikoff Lab and Serge Libert Labs, respectively, within the East Campus Research Facility at Cornell University. Animals were housed in a Specific Pathogen Free facility. Articular cartilage was harvested from the acetabulofemoral joints of 5-day-old UBC mCherry mice after euthanasia by decapitation performed by trained personnel, and chondrocytes were isolated and expanded as previously described^56^**.** Bone marrow-derived MSCs were harvested from 5-week-old PhAM^excised^ mitoDendra2 mice after euthanasia via cervical dislocation by trained personnel, as previously described^57^. Briefly, the tibia, femurs, and humeri and carefully dissected and placed into a petri dish containing alpha minimum essential medium (alpha-MEM, gibco, 1X). The bone marrow is flushed repeatedly into the petri dish using a 23-guage needle attached to a syringe with alpha-MEM. The isolated cells were cultured in alpha-MEM with 2.2 g/L sodium bicarbonate, 15% fetal bovine serum, and 1% Penicillin–Streptomycin (Corning, 100X), pH = 7.2, in 5% CO_2_, 21% O_2_ at 37 °C for 5 days before passaging. Chondrocytes were expanded under either hyperoxic (21% O_2_) or physioxic (5% O_2_) conditions from isolation to passage 3, at which cells were used for experiments. Chondrocytes were plated on 12-well cell culture plates (120,000 cells per well), in DMEM containing either 1 g/L glucose or 0.45 g/L glucose in 21% O_2_ or 5% O_2_ for 1–3 days. At 75% confluence, chondrocytes were stressed by the addition of either a general inflammatory stimulus, IL-1β (Sino Biological, 10 ng/mL), Rot/A (Sigma Aldrich, 0.5 μM, VWR International, 0.5 μM) or another MT-specific stressor, Oligo (Sigma Aldrich O876, 1 μM) for 12 h. During stimulation, chondrocyte media did not contain FBS. After 12 h of stimulation, chondrocytes were rinsed thrice with 1% PBS, fresh chondrocyte media containing FBS was replaced, and passage 3 MSCs were added to each co-culture well in a 10:1 chondrocyte:MSC ratio. After 12 h of co-culture, cells were lifted with 0.25% Trypsin–EDTA (Corning), fixed in 1% formalin, and then resuspended in PBS with 1% bovine serum albumin.

### Flow cytometry

For both equine and murine cells, flow cytometry was performed using a ThermoFisher Attune NxT analyzer located at the Cornell University Biotechnology Resource Center. Single color controls (non-co-cultured cells) were used to create gates that identified each cell type based on size (using forward and side scatter) and fluorescence. An additional gate was created that identified cells with both green and red fluorescence (red^+^/green^+^ cells), indicating MT transfer. These gates were overlayed onto co-cultured samples in FCS Express (equine) or FlowJo (murine) software.

### Confocal imaging of in vitro co-culture models

Imaging experiments utilized a Leica SP5 confocal microscope located at Cornell University. Formalin-fixed equine cells were imaged on a 6-well chambered cover glass slide (see *Equine cell co-culture* model). Live murine cells were imaged longitudinally for up to 9.5 h after initiation of co-culture in a temperature and humidity-controlled chamber outfitted for the microscope.

### Gap junction inhibition

Murine chondrocytes and MSCs were harvested and expanded as above. Chondrocytes were stimulated with Rot/A (Sigma Aldrich, 0.5 μM, VWR International, 0.5 μM) for 12 h before co-culture. MSCs were treated with either CBX (Sigma Aldrich, 100 μM) or Gap27 (Tocris, 100 μM) for 10 h before co-culture, and co-culture wells were also treated with each inhibitor for duration of co-culture. Passage 3 MSCs were added to each co-culture well in a 2:1 chondrocyte:MSC ratio. After 24 h of co-culture, cells were lifted, fixed, and flow cytometry was performed as previously described.

### Chondrocyte gene expression

Chondrocytes were plated on 96 well PCR plates and cultured in physioxia (5% O_2_) or hyperoxia 21% O_2_, in euglycemia (0.45 g/L glucose) or hyperglycemia (1 g/L) and with and without stimulation via Rot/A as above. Briefly, 12–72 h (at ~ 85% confluence) after plating the chondrocytes for experimentation, chondrocytes were either cultured in serum-free media for 12 h (no stim) or stimulated with Rot/A (0.5 µM/0.5 µM) in serum-free media for 12 h. All wells were aspirated of media, rinsed gently with PBS three times, and then trypsinized to remove cells for RNA isolation. Total RNA was isolated with the RNeasy Mini Kit (Qiagen) according to manufacturer protocol. The High-Capacity cDNA Reverse Transcription Kt (ThermoFisher) was used to generate cDNA according to manufacturer protocol. Sample RNA concentration and quality was determined by Nanodrop. Three reactions (10 µL 2 × RT Master mix + 10 µL sample) were performed for each sample. Thermocycler conditions were set at: Step 1—25 °C, 10 min Step 2—37 °C, 120 min Step 3—85 °C, 5 min then Step 4: 4 °C, until samples were removed. For RT-qPCR, sample cDNA was combined with TaqMan Fast Advanced Master Mix (ThermoFisher) according to manufacturer protocol and added to our custom TaqMan Array Plate. The plates were loaded into the ViiA 7 Real-Time PCR system and a Fast Block 96-well experiment was run according to the ViiA instrument user guide. This experiment was replicated four times (n = 4). In the first experiment, technical error in making the hyperglycemic culture medium led to the outliers that were discarded in further analyses.

### Bovine cartilage explant injury and in situ co-culture

Femoral condyle cartilage explants were sterilely harvested from neonatal bovids, obtained post-mortem from a local slaughterhouse, using 6 mm biopsy punches. Explants were cultured in DMEM with 0.45 g/L glucose, 2% sodium pyruvate (Corning), 1% L-glutamine (Corning), 1% fetal bovine serum (R&D systems), 2.5% HEPES (Corning, 1 M), 1% Penicillin–Streptomycin (Corning, 100X), and 0.5% 1% MEM Amino Acids (gibco, 50X) in 5% CO_2_, 21% O_2_. Explants were subjected to injury using a previously validated spring-loaded rapid-impact model^9^. Briefly, the explants were positioned with the articular surface facing up in a PBS filled well and a single, rapid cycle of unconfined axial compression was applied with the impactor. A 10 mm internal spring compression was used, yielding an approximately 17 MPa peak stress and 20 GPa/s peak stress rate as measured by a load cell (50 kHz) at the impactor tip. After impacting, explants were immediately returned to media and incubated overnight at 37 °C, 5% CO_2_, 21% O_2_ prior to MSC seeding. Prior to seeding, cartilage explants were stained with 100 µM carboxyfluorescein diacetate (Thermofisher) for 30 min followed by 30 min rinse and incubation in PBS. Passage 5 bovine MSCs previously transduced with mCherry-mito (Vectalys) were seeded onto the articular surface of the assigned explants in bovine MSC media: DMEM with 1 g/L glucose (Corning, 1X) media with 10% fetal bovine serum (R&D systems), 1% Penicillin–Streptomycin (Corning, 100X), 0.5% Amphotericin B (Corning, 100X) and 0.5% basic fibroblast growth factor (Corning, 1 µg/mL). First, cartilage media was aspirated from the explant wells and 300,000 MSCs in 15 µl media was pipetted onto the articular surface. For 30 min, the 15 µL containing MSCs were incubated on the explants without further addition of cartilage or MSC media to allow maximum seeding. Following this incubation period, cartilage and MSC media was added to the wells in a 1:1 ratio, enough to submerge the explant, and changed every 48 h for the duration of culturing. Confocal imaging studies were performed 4 days after injury and MSC co-culture. Prior to imaging, bovine explants were bisected into hemicylinders using sterile blades. The rectangular portion of the explants were imaged on a Zeiss LSM880 confocal/multiphoton inverted microscope with 10 × and 40 × objectives. Images were acquired in two channel sequential scans (green; 488/498–544 and red; 514/563–663 nm excitation/emission, respectively). All parameters were optimized on the first day of imaging, and the same settings were used on subsequent days. Where MSCs were identified, z stacks were obtained (0.75–5 µm spacing in the z plane). Confocal images were captured and imported into Fiji (ImageJ) for further visualization.

### Murine knee explant and in-situ co-culture with MSCs

Hind limbs were harvested from 10-month-old UBC mCherry mice immediately after euthanasia via cervical dislocation by trained personnel, then skinned and cleaned with sterile PBS. Cuts were made through the lower epiphysis of the femur and upper epiphysis of the tibia to isolate the knee joint, which were placed in PBS with 1% Penicillin/Streptomycin (Corning, 100X). Passage 2 bone marrow derived MSCs were isolated and expanded from PhAM^excised^ mice as previously described^57^. MSCs were lifted and resuspended in PBS at a concentration of 1 × 10^6^ cells/mL. With the aid of a dissecting microscope, ~ 5 µL of this suspension was injected into the knee joints using a 20-gauge, short-beveled needle. Each joint was then placed into the well of a non-tissue culture treated 12-well plate and incubated in media for 8 h at 5% CO_2_, 21% O_2_, 37 °C. Joints were then fixed with 4% paraformaldehyde + 1% cetylpyridinium chloride for 24 h, rinsed 3 times with PBS and decalcified by rocking in 0.1 M EDTA for 7 days. The joints were processed, embedded, and sectioned through the knee joint in either the transverse or sagittal plane using standard protocols. Confocal imaging was performed.

### Quantification and statistical analysis

Data review and statistical analyses for Fig. 1 were performed using GraphPad Prism 9. These data passed the Shapiro–Wilk test for normality (alpha = 0.05). For Fig. 1C, an ordinary one-way ANOVA was performed with post hoc multiple comparisons between group means. For Fig. 1E,F, an unpaired t-test compared the group means.

All data review and analyses for Fig. 2 (panels C and D) were performed using GraphPad Prism 9 and with consultation from the Cornell Statistical Consulting Unit. Normality was assessed using the Shapiro–Wilk or D’Agostino-Pearson omnibus tests (alpha = 0.05). An ordinary one-way ANOVA was performed with post hoc multiple comparisons between group means.

All data review and analyses for Fig. 3 were performed in GraphPad Prism 9. Normality was assessed using the Shapiro–Wilk test (alpha = 0.05). A two-way ANOVA was performed with post hoc multiple comparisons between the control (physiologic conditions) means and the means of hyperoxic and hyperglycemic conditions.

Quantification of gene expression in Fig. 4 was done in Excel where delta cycle threshold (ΔCT) was calculated to evaluate relative transcription levels of the sequence of interest compared to the housekeeping gene (GAPDH) and then ΔΔ CT was calculated by subtracting the ΔCT of the gene of interest in experimental conditions from that of control conditions. These data were log transformed in Excel and then further data review and analyses for were performed in Graphpad Prism 9. Within each gene, equal variance was confirmed by plotting residuals or using the Spearman’s test for heteroscedasticity (*P* > 0.05). Normality of genes GJA1, TIMP1 and SOD2 was determined using the D’Agostino-Pearson omnibus test and the Shapiro–Wilk test. Then, a two-way ANOVA was performed for each gene with post hoc multiple comparisons.

Differences among group means were considered significant when *P* < 0.05. Figures were created in GraphPad Prism 9. Sample size (n) represents the number of biological replicates. No statistical methods were used to predetermine sample size.

### Approval for animal experiments

All experimental protocols were carried out in accordance with animal protocols approved by Institutional Animal Care and Use Committee (IACUC) at Cornell University. All methods, including euthanasia, followed American Veterinary Medical Association (AVMA) and Animal Research: Reporting of In Vivo Experiments (ARRIVE) guidelines.

## Supplementary Information

Supplementary Information.

Supplementary Movie S1.

## Data Availability

All study data are included in the article. The gene expression data discussed in this publication have been deposited in NCBI's Gene Expression Omnibus (GEO)^58^ and is publicly accessible through GEO Series accession number GSE202788 (https://www.ncbi.nlm.nih.gov/geo/query/acc.cgi?acc = GSE202788).
