# Supplementary material for: Mesenchymal stromal cells donate mitochondria to articular chondrocytes exposed to mitochondrial, environmental, and mechanical stress
Source: Sci Rep. 2022 Dec 13;12:21525. doi: 10.1038/s41598-022-25844-5 (PMC9747781; doi:10.1038/s41598-022-25844-5)
Supplement: Supplementary file 1 — Supplementary Information. [file 41598_2022_25844_MOESM1_ESM.pdf]

Supplementary Information for

Mesenchymal Stromal Cells Donate Mitochondria to Articular Chondrocytes Exposed to Mitochondrial, Environmental, and Mechanical Stress

Megan Fahey[1a], Maureen Bennett[1a], Matthew Thomas[a], Kaylee Montney[a], Irene Vivancos-Koopman[a], Brenna Pugliese[a], Lindsay Browning[b], Lawrence J. Bonassar[b], Michelle Delco[a*]

Corresponding author:
Michelle L. Delco, DVM, PhD, DACVS
Email: mld12@cornell.edu

This PDF file includes:

- Supplementary Text
- Figure S1
- Figure S2
- Legend for Figure S1
- Legend for Figure S2
- Legend for Movie S1

Other supplementary materials for this manuscript include the following:

- Movie S1

Supplementary Information Text

MSCs transfer mitochondria (MT) to synoviocytes when injected intra-articularly. Murine MSCs injected intra-articularly can also localize the synovium of the femorotibial (knee) joint to donate MT (Supplementary Fig. S1).

Chondrocytes treated with MSC microvesicles experience improved maximal oxygen consumption (Supplementary Fig. S2)**.**

Supplementary Data

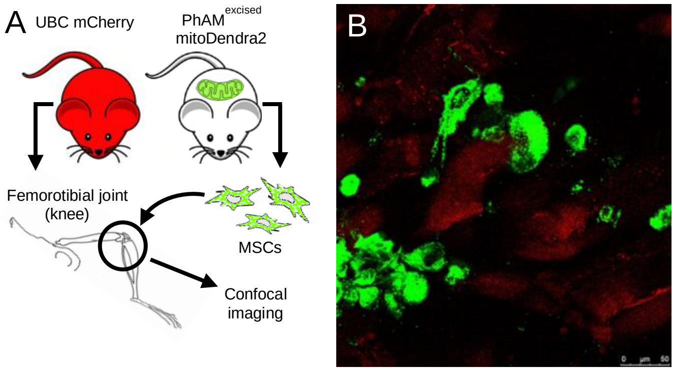

Figure S1. MSCs donate MT to synoviocytes in situ. A) Schematic depicting methods. B) Representative confocal microscopy image of of PhAM mitoDendra2 murine MSCs (green MT) localizing to the synovial membrane and interacting with synoviocytes (red cytoplasm) after intraarticular injection into the knee joint of mCherry mice immediately post-mortem.

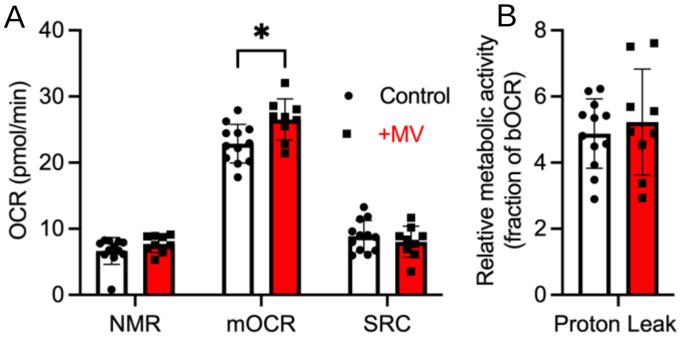

Figure S2. A vehicle of MT donation, MSC-derived microvesicles (MV), improve MT function of treated chondrocytes. A) Equine chondrocyte maximal oxygen consumption rate (mOCR), but not non-mitochondrial respiration (NMR) or spare respiratory capacity (SRC), increases with MV treatment during real-time microscale respirometry (Seahorse MT stress test). B) Proton leak, as a function of relative metabolic activity, does not change with MV treatment.

Movie S1 (separate file). Murine PhAM^{excised} mitoDendra2 MSCs (green MT) and UBC mCherry chondrocytes (red cytoplasm) were imaged longitudinally for up to 9.5 hours after initiation of co-culture in a temperature and humidity-controlled chamber outfitted for the Leica SP5 confocal microscope located at Cornell University. Visualized cellular interactions included MSCs shedding green MT into the extracellular environment and localization of green MT in red chondrocytes.
