# Supplementary figures and images for: Mesenchymal stromal cells donate mitochondria to articular chondrocytes exposed to mitochondrial, environmental, and mechanical stress
Source: Sci Rep. 2022 Dec 13;12:21525. doi: 10.1038/s41598-022-25844-5 (PMC9747781; doi:10.1038/s41598-022-25844-5)

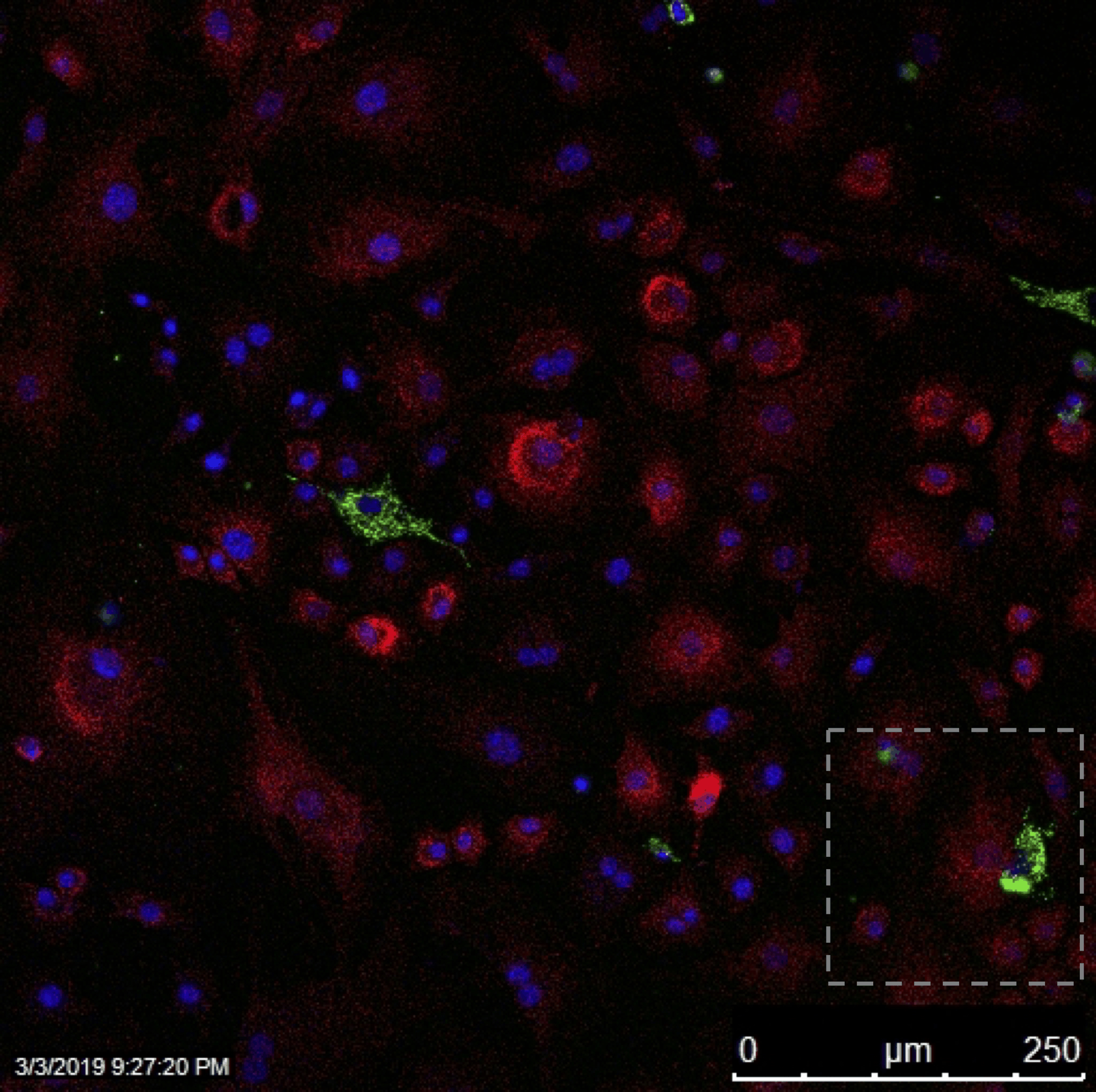

Supplement: Supplementary file 2 — Supplementary Movie S1. [file 41598_2022_25844_MOESM2_ESM.gif]
